# Knowledge, attitude, and practice toward delirium and subtype assessment among Chinese clinical nurses and determinant factors: A multicentre cross-section study

**DOI:** 10.3389/fpsyt.2022.1017283

**Published:** 2023-02-01

**Authors:** Wen Zhou, Qiulan Zheng, Miao Huang, Chuanlai Zhang, Huan Zhang, Li Yang, Taiqin Wu, Xiuni Gan

**Affiliations:** ^1^Department of Nursing, Second Affiliated Hospital of Chongqing Medical University, Chongqing, China; ^2^The Second Department of Nursing School, Chongqing Medical University, Chongqing, China; ^3^Department of Intensive Care Unit, Second Affiliated Hospital, Chongqing Medical University, Chongqing, China

**Keywords:** delirium, delirium subtypes, measurements, nursing assessment, assessment frequency

## Abstract

**Background:**

Delirium, a confused transient state of consciousness, can be divided into hyperactive, hypoactive, mixed, and no motor subtypes, according to different clinical manifestations. Several studies have investigated delirium subtypes in the knowledge dimension, but few studies have investigated delirium subtype in the attitude and practice dimensions. The barriers, knowledge sources, and practice details regarding subtype assessment are unclear.

**Objectives:**

This study had three objectives. First, we planned to investigate the KAP status regarding delirium and subtypes for nurses. Second, we wanted to identify factors affecting clinical nurses' KAP scores. Third, this study expected to explore more details regarding delirium and subtypes assessment, including assessment barriers, assessment instruments, and knowledge sources.

**Methods:**

This multicentre cross-section study was conducted in 10 tertiary hospitals in three provinces, China, from January to April 2022. We investigated 477 nurses from six departments with a high prevalence. The self-developed KAP questionnaire regarding delirium and subtypes assessment had four parts: knowledge, attitude, practice, and source. Its reliability and validity were verified effectively by 2-round Delphi expert consultation.

**Results:**

A total of 477 nurses from the general intensive care unit (ICU), specialty ICU, orthopedics, thoracic surgery, operating room, and geriatrics were 28.3, 22.4, 22.2, 10.5, and 5.2%, respectively. The total KAP score regarding delirium and subtypes assessment was 60.01 ± 6.98, and the scoring rate was 73.18%. The scoring rate for knowledge, attitude, and practice was 58.55, 83.94, and 51.70%, respectively. More than half (54.1%) were unaware of the delirium subtypes assessment instruments. A total of 451 (94.6%) participants recognized the importance of nursing work for delirium prevention. A total of 250 (52.4%) nurses occasionally or sometimes assessed delirium subtypes, and 143 (30.0%) never assessed for delirium subtypes. We found that age, department, technical title, familiarity with delirium, familiarity with delirium subtypes, delirium training, and subtype training affected the total KAP scores. ICU nurses achieved the highest scores.

**Conclusion:**

Chinese nurses' KAP status regarding delirium and subtypes assessment were barely acceptable, and the attitude score was positive, but knowledge and practice needed improvement. Meanwhile, the department was one of the significant KAP factors, and ICU nurses did better in delirium and subtype assessment in knowledge and practice dimension than other departments. Systematic and scientific training processes including subtype content and assessment tools are required. Experience still drives nurses' assessments of delirium and subtype. Adding the delirium assessment into routine tasks should be considered.

## 1. Introduction

Delirium is a confused transient state of consciousness characterized by impaired consciousness, acute onset, fluctuating course, and inattention (1), which has been described, as far back as recorder Roman history (2, 3). Currently, delirium occurs in one-third of hospitalized adults, age 70 and older (4) and in approximately 75, 50, and 90% of ICU mechanical ventilation patients, complex surgery such as hip fracture repair or cardiac surgery, and palliative patients, respectively (5–7). Delirium is also an increased risk predictor of re-intubation and admission to long-term health care facilities, both of which seriously affect patient prognosis (8). Patients with postoperative delirium have an 11 and 17% higher risk of death at 3 months and 1 year postoperatively, respectively (9). For terminal patients, in addition to prolonged hospitalization and increased financial burden, delirium also increases the risk of adverse events (such as falls, stress injuries, and unplanned extubation), accelerates the dying process, and makes it a painful experience for patients, families and caregivers (10, 11).

Delirium has a high incidence among hospitalized patients. Medical staff should identify the condition and each subtype accurately and pay more attention to them (12, 13). According to the Diagnostic and Statistical Manual of Mental Disorders (Fifth Edition) (DSM-V) (14), combined with the clinical manifestations of delirium, scholars have divided delirium into four different clinical subtypes: hyperactive delirium, hypoactive delirium, mixed delirium, and no motor delirium (15, 16). Different subtypes have different incidences and prevalences, clinical outcomes, and nursing priorities (17). Hyperactive delirium is characterized by agitation, anxiety, and removing of external medical devices (e.g., masks, intravenous catheters, drainage tubes, catheters). The overall prevalence of hyperactivity delirium is approximately 15% (18–20). Studies show that the unplanned extubation rate in hyperactive delirium was higher than that in other subtypes, particularly nasogastric and endotracheal intubation (4). The risk of falls is also highest (21). However, the duration of hyperactive delirium, mechanical ventilation time, and ICU hospital stay time are lower than those of other subtypes, while short-term outcomes are better (22). Hypoactive delirium is the most common subtype characterized by confusion, sedation, apathy, unresponsiveness, motor delay, attitudinal withdrawal, and drowsiness (23, 24). The prevalence of cardiac surgery in patients is relatively high at 40% (25). The prevalence of elderly patients after hip surgery is 71% (26). Patients with hypoactive delirium are most likely to have stressful injuries, higher case fatality rates, and relatively poor prognoses (22, 27). Mixed delirium is characterized by fluctuating symptoms of hyperactivity and hypoactivity with intermittent episodes, and the incidence rate is 7.5–54.9% (18, 20, 28, 29). The delirium duration, mechanical ventilation time, ICU days, and total hospital stay time of mixed delirium are more prolonged than those of other subtypes (20, 22, 26). No motor subtype delirium is the altered state of consciousness without psychomotor disturbance (such as hyperkinesia or bradykinesia) (4, 16, 30, 31).

Previous studies indirectly suggested a variation in the ability of health care professionals to assess each subtype. A systematic review showed that hypoactive delirium was missed in two-thirds of adult ICU patients (32). Hyperactivity is more likely to be noticed by health care providers, while hypoactivity is more likely to be overlooked (33). This means that the ability of Chinese nurses to identify delirium subtypes needs to be improved, just as Sun's research shows that nurses identified only 17.6% of hypoactive deliriums (18). There is a significant correlation between human resources investment and patient care safety outcomes, and a nursing shortage exists in China (34, 35). Thus, nursing efficiency is key, and educating and training nurses to identify and manage patients according to subtypes will help them apply the appropriate interventions and meet the requirements of precision medicine and precision nursing (36–38). As the first direct caregivers of in-hospital patients, nurses need to apply delirium knowledge, accurately identify subtypes, and manage each accordingly to improve delirium diagnosis and management (18). Nurses' knowledge, attitudes, and practices (KAP) influence the successful management of each subtype.

However, the KAP level of nurses regarding delirium subtypes is unclear. Several studies have investigated delirium subtypes in the knowledge dimension, but few studies have investigated delirium subtype in the attitude and practice dimensions (39, 40). Additionally, previous studies mostly investigated the staff from a single department, such as the ICU (39). Few studies have explored the differences in delirium assessment ability among departments with high incidences of delirium. Thus, this study aims to address the unclear status of the delirium subtype assessment among hospital departments. Based on several delirium guidelines (12, 13, 41, 42), this research focused on the current KAP status regarding delirium subtypes among the nursing staff of different departments with high delirium rates using a self-administered questionnaire with the theoretical guidance of the Knowledge-Attitude-Practice Model (43). We also explored the influencing factors of KAP status, which could provide suggestions for future studies related to delirium and delirium subtypes. We also explored the barriers to delirium and subtype assessment, surveyed the knowledge sources, and detailed delirium practices.

## 2. Methods

### 2.1. Study design and participants

An online cross-section study was conducted using a convenience sampling method targeting nurses from clinical departments with a high incidence of delirium. In this study, we investigated the following departments: the general intensive care unit, specialty intensive care unit, thoracic and cardiac surgery department, orthopedics (joint surgery) department, operating room and geriatric department in 10 hospitals in mainland China. Each hospital is a large grade A tertiary hospital (the highest level in Chinese hospitals) or a teaching hospital affiliated with one of three universities Chongqing Medical University, University of Chinese Academy of Science, Army Medical University. This study took place from January to April 2022 and used a convenience sampling method. All registered nurses from eligible units with at least 1 year of experience were invited to participate voluntarily in the survey. The exclusion criteria were as follows: (1) nurses who were not working in a clinically responsible nursing position when the survey was conducted (i.e., on sick leave or maternity leave); and (2) nurses who were not independently responsible for their patients, such as interns and trainees. The results are reported in line with the Checklist for Reporting the Results of Internet E-Surveys (CHERRIES) to ensure the quality of this study, as shown in Supplementary material 1.

### 2.2. Development of the delirium subtype KAP questionnaire

Knowledge-attitude-practice (KAP) theory has been listed as one of the four most influential behavioral intervention theories by the American Center for Disease Prevention (44, 45). It is one of the most frequently used study models in health-seeking behavior research to collect information on what is known, believed, and done in a particular topic among a specific population (46). Knowledge is usually evaluated to see the extent to which knowledge corresponds to biomedical concepts (47). People's reported knowledge that deviates from biomedical concepts is usually termed “beliefs” (48). Attitude is defined as “a learned predisposition to think, feel and act in a particular way toward a given object or class of objects” (49). Practices in KAP surveys usually enquire about the use of preventive measures or different health care options (50). Knowledge, attitude and practice constitute a triad of interactive factors characterized by dynamism and unique interdependence. Emphasis for each component of the triad is placed on the value of ethical conduct in applying the component to patient care (51). When people acquire relevant knowledge and respond positively, they gradually form beliefs. With relevant knowledge and positive beliefs, it is possible to adopt a positive attitude and change behaviors (52). KAP theory is widely used in all aspects of Chinese nursing research, such as studies on nursing practice, management and education, with the aim of improving nursing behaviors (53, 54).

Based on the Knowledge-Attitude-Practice Model theory, the clinical nurses' delirium subtype KAP questionnaire was formed by reviewing several guidelines (12, 13, 41, 42). To explore more details, we designed a KAP questionnaire with several types of questions: true or false, single-choice, multiple-choice, scales (measure on a 5-point Likert scale), and fill-in-the-blank. There was only one true answer in each single-choice question, and more than one true answer for each multiple-choice question. The questionnaire consisted of 37 items in four parts: knowledge dimension (11 items, including one true or false question and ten multiple-choice questions), attitude dimension (16 items, including 10 scales, five multiple-choice questions and one single-choice question), practice dimension (five items, including four single-choice questions and one multiple-choice question), and knowledge sources dimension (five items, including one scale, one single-choice question and three multiple-choice questions). We also designed six ancillary questions about practice dimensions and two ancillary questions of knowledge sources. The questionnaire is shown in Supplementary material 2.

Moreover, 10 experts were invited into the Delphi consultation when the draft of the questionnaire was developed. Their research fields were critical care medicine and nursing, anesthesia nursing, geriatric nursing, and nursing management. The experts were all familiar with delirium management and were asked to consider the rationality and importance of the items and give their opinion on each item. The questionnaire was initially developed through two rounds of the Delphi expert correspondence method when the experts reached a basic agreement. The consistency of 10 experts from seven provinces was evaluated by the mean value and coefficient of variation (CV) of the importance score and the rationality score. The mean value reflected the importance and rationality of each item. The CV reflected the measures of dispersion of each item. If the CV was lower than 0.25, it was considered acceptable. The experts scored the importance and rationality of each item according to a 5-point Likert scale, in which points 1–5 represented increasing levels of rationality or importance. Finally, researchers finalized the questionnaire based on the experts' opinions.

The knowledge dimension included 11 items (eight delirium items, three subtype items). Delirium items included the definition, outcomes, high-risk factors, high-risk patients, clinical features, assessment instruments, management measures, and strategies; subtype items included the clinical manifestations, poor outcomes, and assessment instruments of each delirium subtype. The knowledge dimension was worth a total of 22 points, with two points for correct responses and zero points for incorrect responses in true-or-false question, two points for all correct multiple-choice questions, one point for partially correct responses, and zero points for incorrect responses.

The attitude dimension scored nurses' attitudes regarding the importance, assessment, responsibility, and training of delirium and subtypes, with scores from 1 to 5 points representing approval of the items from “strongly disagree” to “strongly agree” for a total of 50 points. Three multiple-choice questions in the attitude dimension were used to investigate the barriers to assessment. Nurses were asked to self-assess the quality of delirium assessment and delirium subtype assessment in their own departments in a single-choice question. One multiple-choice test was used to investigate the habitual expressions of each subtype. We also investigated the requirements of delirium subtype assessment tools.

In the practice dimension, a Likert 5-point scale was used to explore the frequency of nurses' delirium and subtype assessment, with 1–5 points representing “never” to “always”, for a total of 10 points. Multiple-choice questions explored the collaborative behavior and documented behavior of delirium and subtypes. We also surveyed the most common subtype in daily work. Six ancillary questions were used to explore the details of nurses' recording behavior, assessment instruments that nurses used, and the reasons for their behavior.

The knowledge source dimension included the source and training needs regarding delirium subtypes. There were two ancillary questions regarding training. This part was an addition to the KAP survey, so we did not include it in the total score calculation. It was only designed to elicit suggestions for future research, such as developing training programs and quality improvement.

### 2.3. Reliability and validity of the delirium subtype KAP questionnaire

The reliability of the delirium subtype KAP questionnaire was measured by Cronbach's alpha coefficient and discussed by a small focus group. The internal consistency reliability test included all scoring questions and two single-choice questions in the practice dimension using a 5-point Likert scale. The overall Cronbach's alpha coefficient was 0.860, which was acceptable. We invited four professionals (three head nurses and one physician) to join our focus group. All focus group members (four head nurses, one doctor, three specialist nurses, and five graduate students) were asked to make suggestions on the structure of the questionnaire and the readability of each item. They were asked to consider whether the expression of each item was understandable and ambiguous. All members agreed that all items were easy to understand and that the structure was reasonable.

The content validity of the questionnaire was tested using the Delphi method. The response rate of experts reflected the degree of expert positivity to the delirium subtypes KAP questionnaire. The response rate of experts for the two rounds was 83.3% (12 invited, 10 agreed) and 100% (10 invited, 10 agreed). The mean value of the importance score of each item was 4.40–5.00, and the CV was 0.00–0.15. The mean value of the rationality score of each item was 4.60–5.00, and the CV was 0.00–0.16. These results indicated that experts agreed on the rationality and importance of the delirium subtype KAP questionnaire. Additionally, we invited 26 clinical nurses to participate in the preliminary survey and required them to make suggestions regarding the expression of each item. The final version of the KAP questionnaire was affirmed following discussion of the focus group's input by researchers. As a result, we believe the KAP questionnaire for delirium and subtypes assessment is reliable and valid.

### 2.4. Data collection

The data collection instruments had two parts: the demographic information of the participants and a delirium subtype KAP questionnaire. Demographics consisted of 11 questions, including sex, age, clinical working years, work department, education, technical title, position, familiarity with the field of delirium and subtypes, and training experience in delirium and subtypes. Familiarity was scored on a 5-point Likert scale, with points 1–5 representing increased familiarity. A dichotomous variable collected training experience. The delirium subtypes KAP questionnaire was developed above.

Data were collected from January to April 2022. We entered the demographic content and the delirium subtype KAP questionnaire content into the *Wenjuanxing* platform, a free and open online survey website. All content was eventually presented as an electronic questionnaire which was given a unique QR code. All questions were set as mandatory to avoid missing items, except eight ancillary questions in the practice dimension and the knowledge source dimension. All participants were required to complete 11 demographic questions and 37 essential questionnaire items. If participants selected several sections connected with an ancillary question, they were asked to complete the additional problem. We sent the QR code to the head nurse in each department by e-mail, WeChat, or in person. The inclusion and exclusion criteria were clarified. All participants provided informed consent before they began the survey. The investigation was confidential and anonymous. Respondents were allowed to change their responses using a “Back” button at the bottom of each page, which was a function provided by the *Wenjuanxing* platform. Participants could end the survey at any time if they closed the link or did not submit the survey, and their data would not be retained. The questionnaire could not be revoked after submission.

### 2.5. Data analysis

Data entry and analysis were performed using SPSS 26.0. Descriptive statistics of each item or selection included frequencies and percentages. Continuous variables, such as scores, were measured as the means (M) ± standard deviations (SD). Categorical variables were expressed as frequencies and percentages in demographics. *T*-tests and *F*-tests were used for one-way analysis. Pearson correlation analysis was used to investigate the correlation between general factors and scores by the Pearson correlation coefficient (*r*). Multivariate stepwise linear regression models were applied to examine the factors influencing the scores. *P* < 0.05 was considered statistically significant.

### 2.6. Ethical aspects

The Ethics Committee approved the study of the Second Affiliated Hospital of Chongqing Medical University (Ethical Approval Number: 2022-11). All participants volunteered for this survey. We provided the introduction of this survey and informed consent on the first page of the survey accessed by scanning the QR code. If participants were interested and want to look through the survey questionnaire, they would tick the button on the first page, which was written in “I agree to participate in this research of my own volition”. The *Wenjuanxing* platform recorded their informed consent automatically, which meant the participants had made the informed consent. Then they would enter the formal survey. The survey promised autonomy, anonymity, and no harm, according to the Declaration of Helsinki. The survey did not include any patients or animals during the research process.

## 3. Results

### 3.1. Participants' characteristics

We received 492 responses, of which 477 were valid, for a 98.0% response rate. The number of completed surveys from the general ICU, specialty ICU, orthopedics, thoracic surgery, operating room, and geriatrics were 28.3, 22.4, 22.2, 10.5, and 5.2%, respectively. The familiarity level was measured on a 5-point Likert scale, with points 1–5 indicating “very unfamiliar” to “very familiar”. The familiarity level with delirium was 3.34 ± 0.83, and delirium subtype familiarity was 2.95 ± 0.85. A total of 182 (38.2%) and 198 (41.5%) had knowledge of delirium at a level “very familiar” (four point) and “moderately familiar” (three point), respectively. Sixty-three (13.2%) evaluated themselves at a level of “slightly familiar” (two point). Conversely, 204 (42.8%) participants and 138 (28.9%) had knowledge of delirium subtypes at a level of “moderately familiar” (three point) and “slightly familiar” (two point). Only 28 (5.9%) participants were extremely familiar with delirium and 13 (2.7%) with subtypes. A total of 185 (38.8%) nurses attended training on delirium, but only 73 (15.3%) attended training on delirium subtypes. Details of the demographic characteristics are shown in the first Two columns of Table 1.

**Table 1:** Demographic characteristics of participants and one-way ANOVA analysis of delirium and subtype assessment for clinical nurses (*n* = 477).

	**Number (percent)**	**Knowledge score**		***p***	**Attitude score**		***p***	**Practice score**		***p***	**Total score**		***p***
Sex			0.258^a^	0.797		0.553^a^	0.58		2.162^a^	0.031		1.142^a^	0.254
Male	50 (10.5%)	12.98 ± 3.03			42.34 ± 5.21			5.76 ± 1.99			61.08 ± 7.50		
Female	247 (89.5%)	12.87 ± 2.80			41.92 ± 5.03			5.10 ± 2.06			59.89 ± 6.91		
Age			4.406^b^	0.005		1.814^b^	0.144		5.855^b^	0.001		4.452^b^	0.004
20–29	220 (46.1%)	13.21 ± 2.83			42.41 ± 5.50			5.48 ± 2.08			61.10 ± 7.26		
30–39	221 (46.4%)	12.67 ± 3.05			41.42 ± 4.39			5.03 ± 2.02			59.11 ± 6.78		
40–49	30 (6.2%)	12.77 ± 5.17			42.87 ± 5.57			4.17 ± 1.52			59.80 ± 10.84		
50–59	6 (1.3%)	9.50 ± 2.82			41.33 ± 5.04			3.50 ± 2.06			54.33 ± 6.98		
Clinical working years			2.682^b^	0.046		0.450^b^	0.717		2.090	0.101		1.700^b^	0.166
Less than 3 years	104 (21.8%)	13.04 ± 2.74			42.11 ± 4.87			5.45 ± 1.89			60.60 ± 6.60		
3–10 years	212 (44.4%)	13.17 ± 2.63			41.98 ± 5.13			5.26 ± 2.12			60.41 ± 7.00		
11–15 years	95 (19.9%)	12.25 ± 2.65			41.49 ± 5.20			4.92 ± 2.02			58.66 ± 6.50		
≥15 years	66 (13.8%)	12.60 ± 3.58			42.38 ± 4.86			4.77 ± 2.13			59.76 ± 7.94		
Work department			5.163^b^	<0.001		3.579^b^	0.003		13.573^b^	<0.001		9.901^b^	<0.001
General ICU	135 (28.3%)	13.47 ± 2.69			42.27 ± 4.76			6.11 ± 1.71			61.85 ± 6.07		
Special ICU	107 (22.4%)	13.23 ± 2.58			42.41 ± 5.31			5.39 ± 2.25			61.04 ± 7.17		
Orthopedics	106 (22.2%)	12.78 ± 3.03			42.53 ± 4.77			4.86 ± 2.16			60.17 ± 6.67		
Cardio-thoracic surgery	54 (11.3%)	12.76 ± 2.12			42.22 ± 4.65			4.54 ± 1.66			59.52 ± 5.26		
Anesthesiology	50 (10.5%)	11.48 ± 3.41			40.22 ± 5.06			3.98 ± 1.82			55.68 ± 7.89		
Geriatrics	25 (5.2%)	11.68 ± 2.50			39.00 ± 5.94			4.12 ± 1.33			54.80 ± 7.58		
Education			2.067^b^	0.128		2.500^b^	0.083		0.389^b^	0.678		2.087^b^	0.125
Associate	48 (10.1%)	12.10 ± 3.48			41.46 ± 4.74			5.40 ± 2.11			58.96 ± 7.94		
Bachelor	413 (86.6%)	12.96 ± 2.72			41.92 ± 5.09			5.13 ± 2.06			60.02 ± 6.92		
Master and doctor	16 (3.4%)	13.13 ± 3.07			44.62 ± 4.13			5.31 ± 2.18			63.06 ± 4.31		
Technical title			1.337	0.262		2.745	0.043		4.396	0.005		4.428	0.004
Primary	119 (24.9%)	13.19 ± 2.71			42.26 ± 4.71			5.70 ± 1.96			61.15 ± 6.52		
Primary (supervisor)	194 (40.7%)	12.97 ± 2.59			42.41 ± 4.69			5.14 ± 2.07			60.53 ± 6.44		
Moderate	142 (29.8%)	12.53 ± 3.12			40.97 ± 5.83			4.79 ± 2.00			58.29 ± 7.90		
Senior	22 (4.6%)	12.68 ± 3.24			42.86 ± 3.44			4.95 ± 2.44			60.50 ± 5.79		
**Position**													
Duty nurse			−1.537^a^	0.126		−1.143^a^	0.254		−2.628^a^	0.009		−2.301^a^	0.022
Yes	330 (69.2%)	13.03 ± 2.52			42.14 ± 5.05			5.33 ± 2.09			60.50 ± 6.63		
No	147 (30.8%)	12.55 ± 3.39			41.57 ± 5.03			4.80 ± 1.96			58.92 ± 7.60		
Group leader			−2.221^a^	0.027		−0.522^a^	0.602		0.978^a^	0.329		−0.983^a^	0.326
Yes	70 (14.7%)	13.57 ± 2.44			42.26 ± 5.11			4.94 ± 2.17			60.77 ± 6.75		
No		12.76 ± 2.87			41.92 ± 5.03			5.20 ± 2.04			59.88 ± 7.01		
Clinical instructor	407 (85.3%)		0.193^a^	0.847		0.590^a^	0.556		0.751^a^	0.453		0.726^a^	0.468
Yes	74 (15.5%)	12.82 ± 2.69			41.65 ± 5.11			5.00 ± 1.97			59.47 ± 6.65		
No	403 (84.5%)	12.89 ± 2.85			42.02 ± 5.03			5.20 ± 2.08			60.11 ± 7.04		
Specialist nurse			1.180^a^	0.239		−0.003^a^	0.998		0.487^a^	0.627		0.619^a^	0.537
Yes	63 (13.2%)	12.49 ± 3.07			41.97 ± 4.80			5.05 ± 2.16			59.51 ± 7.86		
No	414 (86.8%)	12.94 ± 2.78			41.97 ± 5.08			5.18 ± 2.05			60.09 ± 6.84		
Head nurse			0.583^a^	0.560		−0.949^a^	0.343		−0.018^a^	0.985		−0.455^a^	0.649
Yes	29 (6.1%)	12.59 ± 3.10			42.83 ± 4.58			5.17 ± 2.32			60.59 ± 6.88		
No	448 (93.9%)	12.90 ± 2.80			41.91 ± 5.07			5.17 ± 2.05			59.98 ± 6.99		
Familiarity of delirium			3.290^b^	0.011		7.402^b^	<0.001		34.815^b^	<0.001		18.256^b^	<0.001
Extremely	28 (5.9%)	12.86 ± 3.76			44.07 ± 4.40			7.36 ± 1.89			64.29 ± 5.89		
Very	182 (38.2%)	13.28 ± 2.35			42.93 ± 4.11			5.97 ± 1.93			62.18 ± 5.40		
Moderately	198 (41.5%)	12.83 ± 2.87			41.45 ± 5.15			4.63 ± 1.81			58.91 ± 6.83		
Slightly	63 (13.2%)	12.14 ± 2.86			40.46 ± 5.53			3.68 ± 1.48			56.29 ± 7.47		
Not at all	6 (1.3%)	10.33 ± 6.02			35.83 ± 11.60			3.67 ± 1.97			49.83 ± 15.46		
Familiarity with delirium subtypes			1.331^b^	0.258		4.216^b^	0.002		29.886^b^	<0.001		11.425^b^	<0.001
Extremely	13 (2.7%)	12.86 ± 3.76			44.07 ± 4.40			7.36 ± 1.89			64.29 ± 5.89		
Very	110 (23.1%)	13.28 ± 2.35			42.93 ± 4.11			5.97 ± 1.93			62.18 ± 5.40		
Moderately	204 (42.8%)	12.83 ± 2.86			41.45 ± 5.15			4.63 ± 1.81			58.91 ± 6.83		
Slightly	138 (28.9%)	12.14 ± 2.86			40.46 ± 5.53			3.68 ± 1.48			56.29 ± 7.47		
Not at all	12 (2.5%)	10.33 ± 6.02			35.83 ± 11.60			3.67 ± 1.97			49.83 ± 15.46		
Training of delirium			2.911^a^	0.004		1.816^a^	0.070		6.113^a^	<0.001		4.297^a^	<0.001
Yes	185 (38.8%)	13.35 ± 2.59			42.49 ± 4.56			5.86 ± 1.99			61.71 ± 6.03		
No	292 (61.2%)	12.59 ± 2.92			41.63 ± 5.30			4.72 ± 1.99			58.94 ± 7.32		
Training of delirium subtypes			0.873^a^	0.331		1.248^a^	0.213		6.835^a^	<0.001		3.258^a^	<0.001
Yes	73 (15.3%)	13.18 ± 2.82			42.64 ± 4.53			6.62 ± 1.93			62.44 ± 6.18		
No	404 (84.7%)	12.83 ± 2.82			41.84 ± 5.12			4.90 ± 1.98			59.58 ± 7.02		

a *T*-test.

b *F*-test.

### 3.2. Knowledge and knowledge sources of nurses regarding delirium and subtype assessment

**Figure 1:**
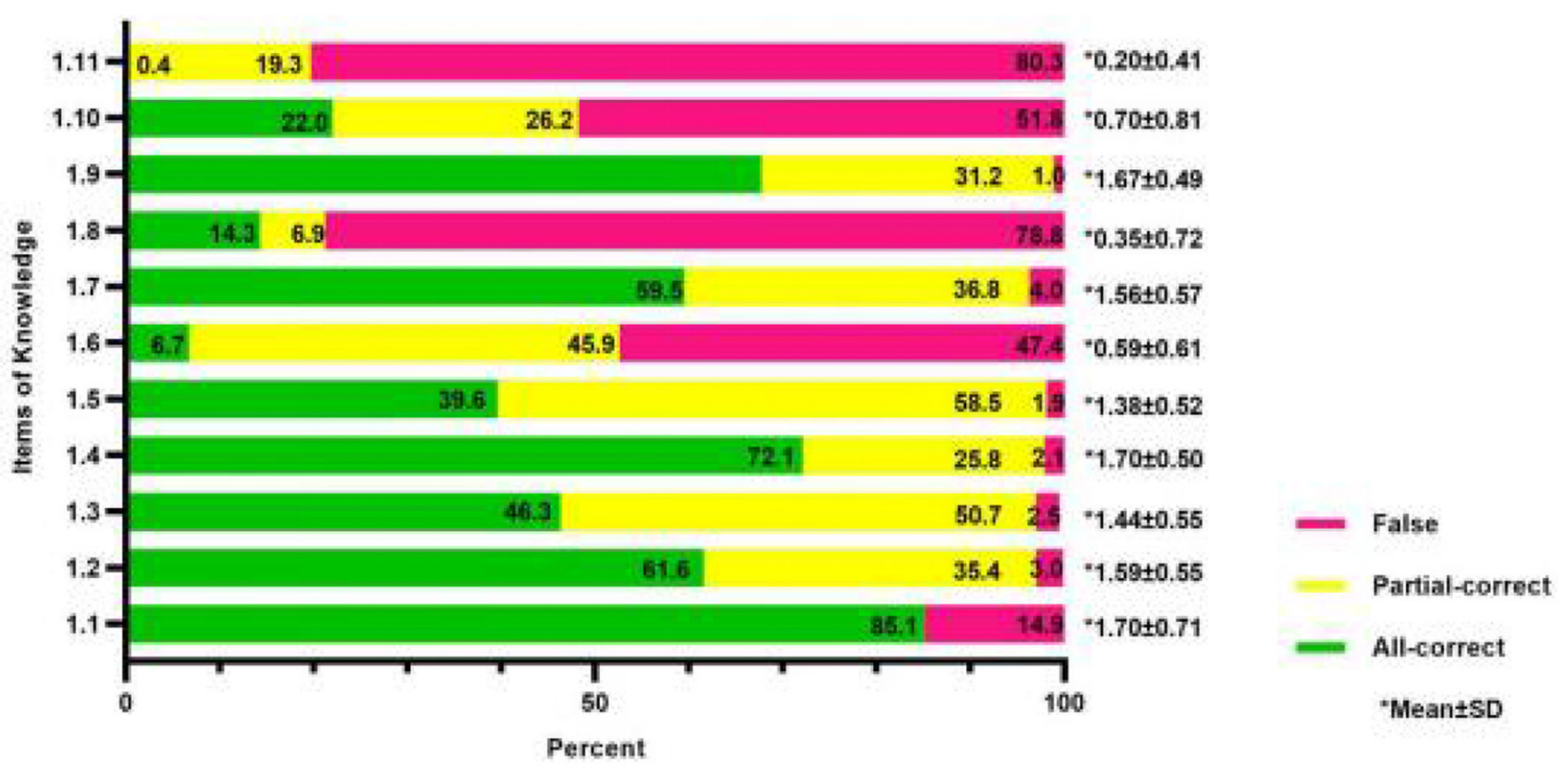
Knowledge score of delirium and subtype assessment for clinical nurses (*n* = 477).

The total score of 477 participants in the knowledge part was 12.88 ± 2.82, and the scoring rate was 58.55%. The percentages of participants with all-correct, partial-correct, and false choices are shown in Figure 1. The three entries with the highest number of all-correct, partial-correct, and false responses are shown in Table 2. Among the responses for clinical manifestations of delirium, 40.7% of nurses chose both increased and decreased activity, with up to 37.5% selecting only increased activity and 5.5% selecting only decreased activity. A total of 358 (75.1%) nurses considered that restraint was an effective way to prevent delirium. Each of the false options was selected by one-third of participants in poor outcomes of subtypes. Additionally, more than half (54.1%) were unaware of the delirium subtype assessment instruments.

**Table 2:** The three items with the highest number of all-correct, partial-correct, and false in knowledge section.

	**Number (percent)**	**Mean ±SD**
**Highest number of all-correct**		
1.1. Definition of delirium	406 (85.12%)	1.70 ± 0.71
1.4. High-risk factors of delirium	344 (72.12%)	1.70 ± 0.50
1.9. Definition of delirium subtype	323 (67.71%)	1.67 ± 0.49
**Highest number of false**		
1.11. Assessment tools of delirium subtype	383 (80.29%)	0.20 ± 0.41
1.8. Prevention measures of delirium	376 (78.83%)	0.35 ± 0.72
1.10. Poor outcomes of delirium subtype	247 (51.78%)	0.70 ± 0.81
**Highest number of partial-correct**		
1.5. Clinical significance of delirium	279 (58.49%)	1.38 ± 0.52
1.3. People at high risk of delirium	242 (50.73%)	1.44 ± 0.55
1.6. Assessment tools of delirium	219 (45.91%)	1.56 ± 0.57

**Figure 2:**
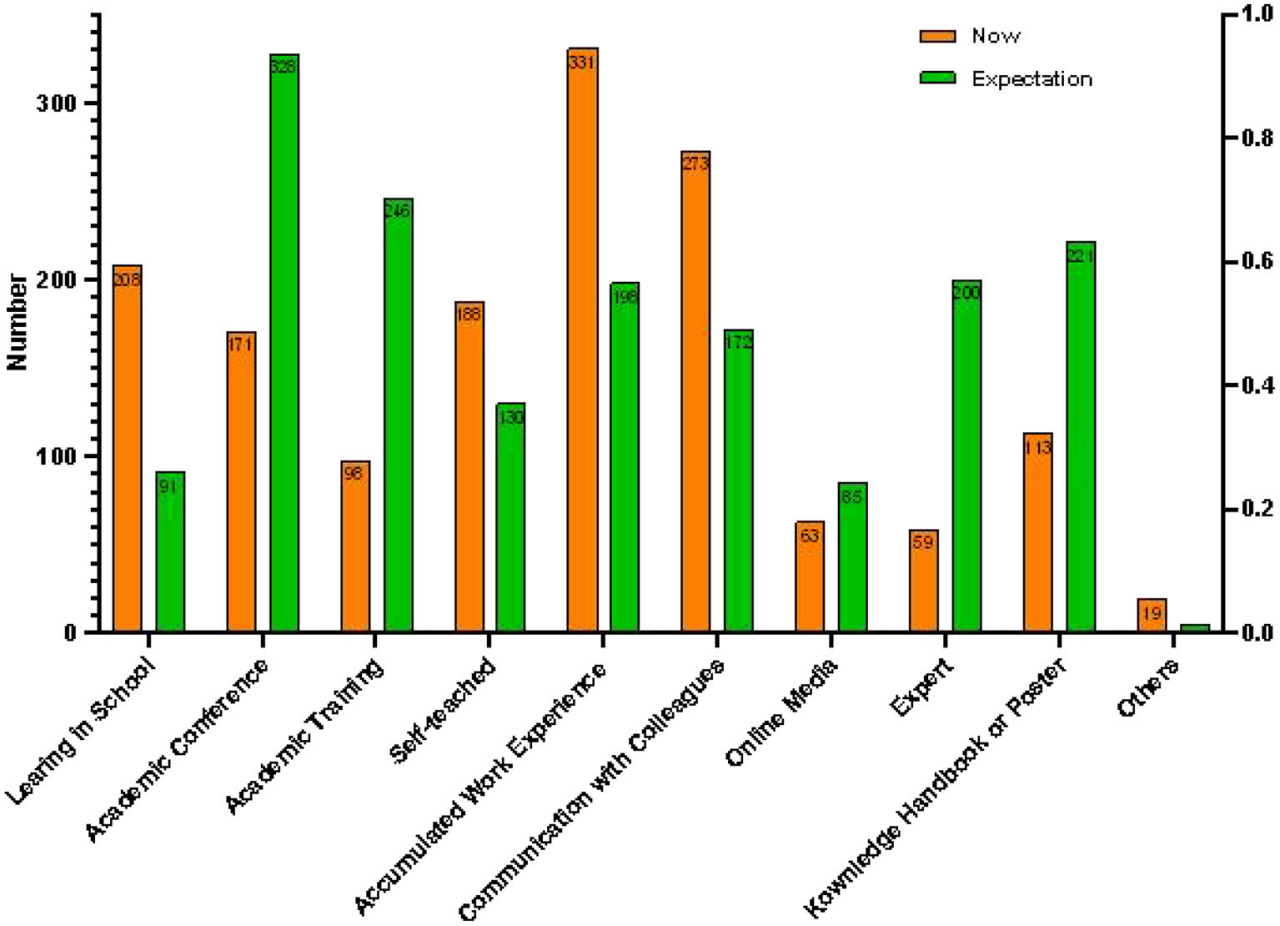
Primary knowledge sources at present and the most desired in the future for clinical nurses.

The results of the knowledge source showed that primary sources were “the accumulation of work experience” (69.4%), “communication among colleagues” (57.2%), and “knowledge learned in school” (43.6%). Findings also showed that nearly 40% are taught by themselves because of work necessity. Systematic and scientific training and learning were lacking, which were also desired in the future. The primary knowledge sources at present and the most desired future sources shown in Figure 2.

### 3.3. Attitudes of nurses regarding delirium and subtype assessment

**Figure 3:**
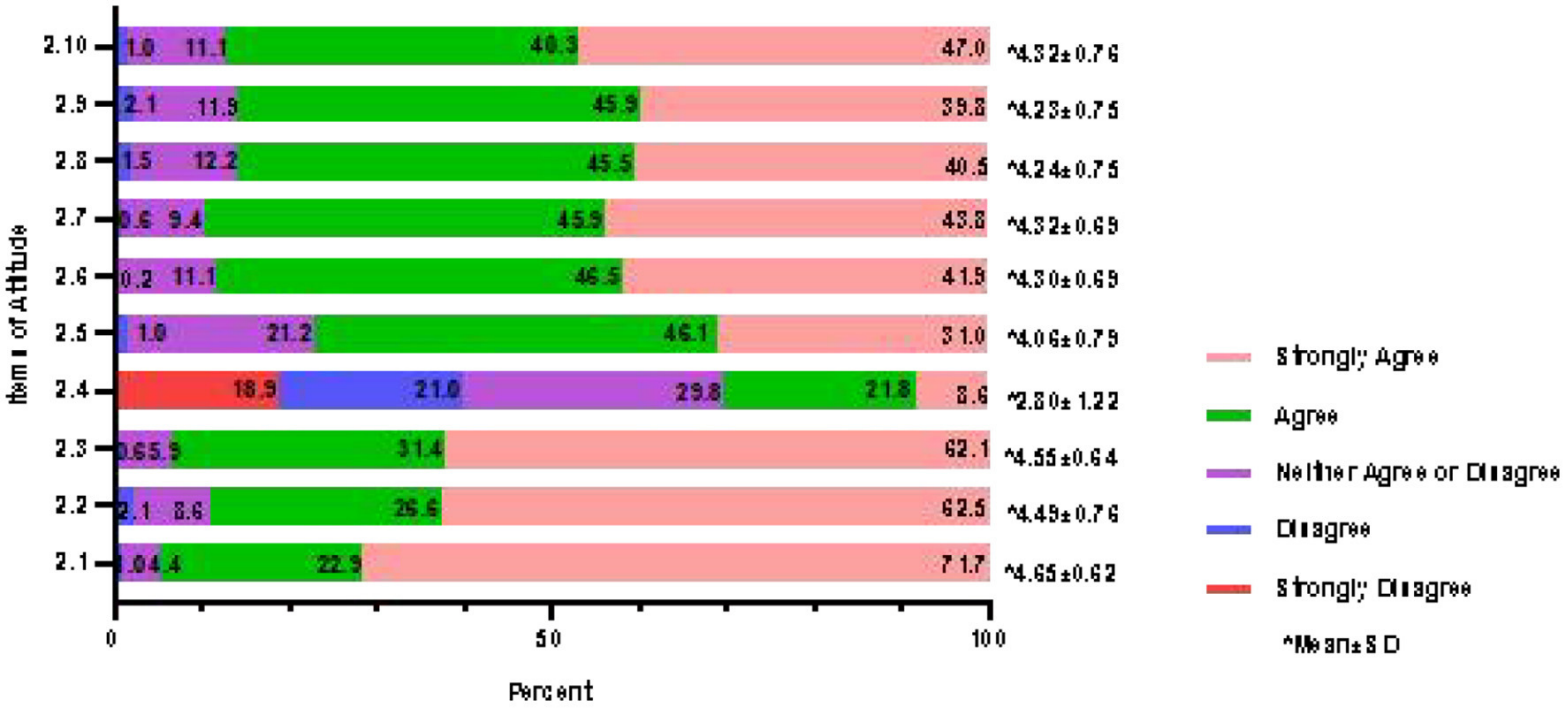
Attitude score of delirium and subtype assessment for clinical nurses (*n* = 477).

The total score of 477 nurses for positive attitude was 41.97 ± 5.04, and the scoring rate was 83.94%. The percentages of the 10 scale questions are shown in Figure 3 (except for Item 2.4, the percentage of participants who chose “completely disagree” for all items was ≤ 0.6%, so they are not shown in the figure). The survey demonstrated that the overall attitude of clinical nurses toward delirium and subtype assessment was positive. A total of 451 (94.6%) participants recognized the importance of nursing work for delirium prevention. The frequency of subtypes encountered in daily work was ranked from high to low hyperactive, mixed, hypoactive, and no motor subtype. The details regarding barriers are shown in Table 3. It is worth noting that insufficient knowledge of delirium and its subtypes accounted for the vast majority. A total of 305 nurses (63.94%) believed that delirium assessment was currently immature and subtype assessment was not performed at all. When nurses evaluated delirium assessment work in their own departments, a quarter (25.16%) said their clinical departments had never assessed delirium, while 29.14% thought that the departments had unsatisfactory delirium assessment, with 28.72% reporting moderate work and 16.98% reporting excellent work.

**Table 3:** Barriers of delirium and subtype assessment for clinical nurse (*n* = 477).

	**Number (percent)**
**Individual-level impairment factors in delirium assessment**	
The insufficient knowledge base of delirium	423 (88.68%)
Insufficient mastery of delirium assessment methods	394 (82.60%)
Insufficient proficiency in the use of delirium assessment scales	374 (78.41%)
Lack of time for delirium assessment due to nurses' busy clinical schedule	327 (68.55%)
Nurses are not confident in their ability to assess delirium and do not trust the results of their assessment	285 (59.75%)
The increased workload associated with delirium assessment	256 (53.67%)
Insufficient cooperation between nurses and physicians	236 (49.48%)
**Organizational level impairment factors in delirium assessment**	
The department/hospital does not have a process protocol for delirium assessment	347 (72.75%)
No training on delirium assessment in the department/hospital	345 (72.33%)
Delirium assessment is not routinely performed in the unit	328 (68.76%)
Delirium assessment tools are not available in the department	293 (61.43%)
Inadequate human resource allocation in the department	237 (49.69%)
Others	22 (4.61%)
**Impairment factors in delirium subtype assessment**	
Nurses have inadequate knowledge of delirium subtype and assessment methods	350 (73.38%)
Lack of objective delirium subtype assessment tools for nurses	322 (67.51%)
Delirium assessment is still immature, and subtype assessment is not conducted at all	305 (63.94%)
Nurses' clinical workload is busy, and delirium subtype assessment increases nurses' workload	268 (56.18%)
The department/hospital is not currently focused on delirium subtype assessment	230 (48.22%)
The clinical presentation of each delirium subtype is not very different	224 (46.96%)
The management of delirium subtype does not differ significantly from one another	217 (45.49%)
The prognostic impact of each subtype of delirium does not differ significantly	172 (36.06%)
Others	12 (2.52%)

### 3.4. Practice of nurses regarding delirium and subtype assessment

**Figure 4:**
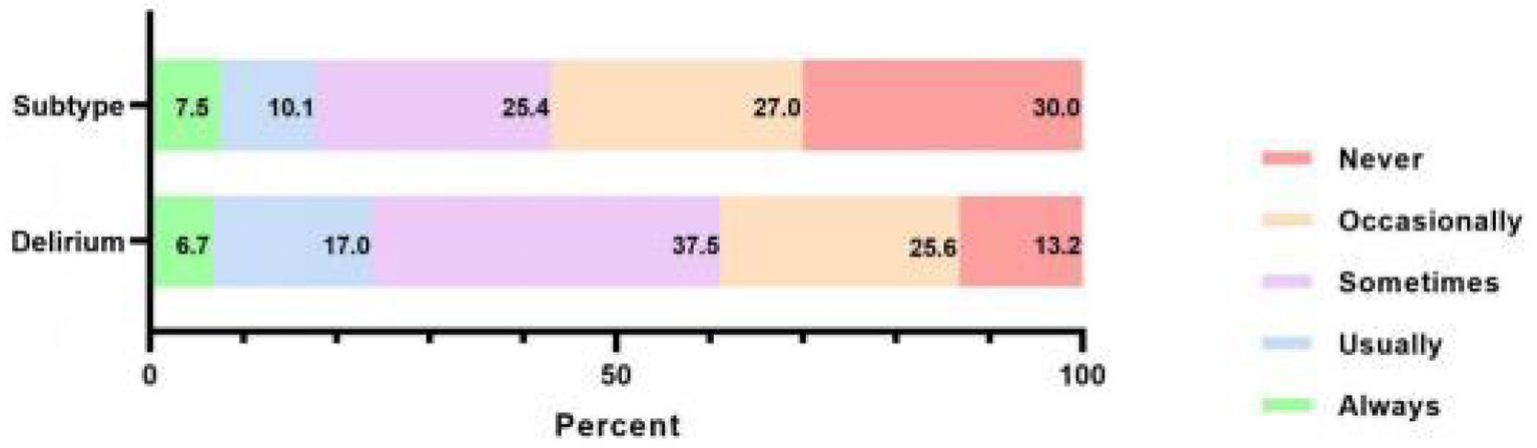
Practice score of delirium and subtype assessment for clinical nurses (*n* = 477).

**Figure 5:**
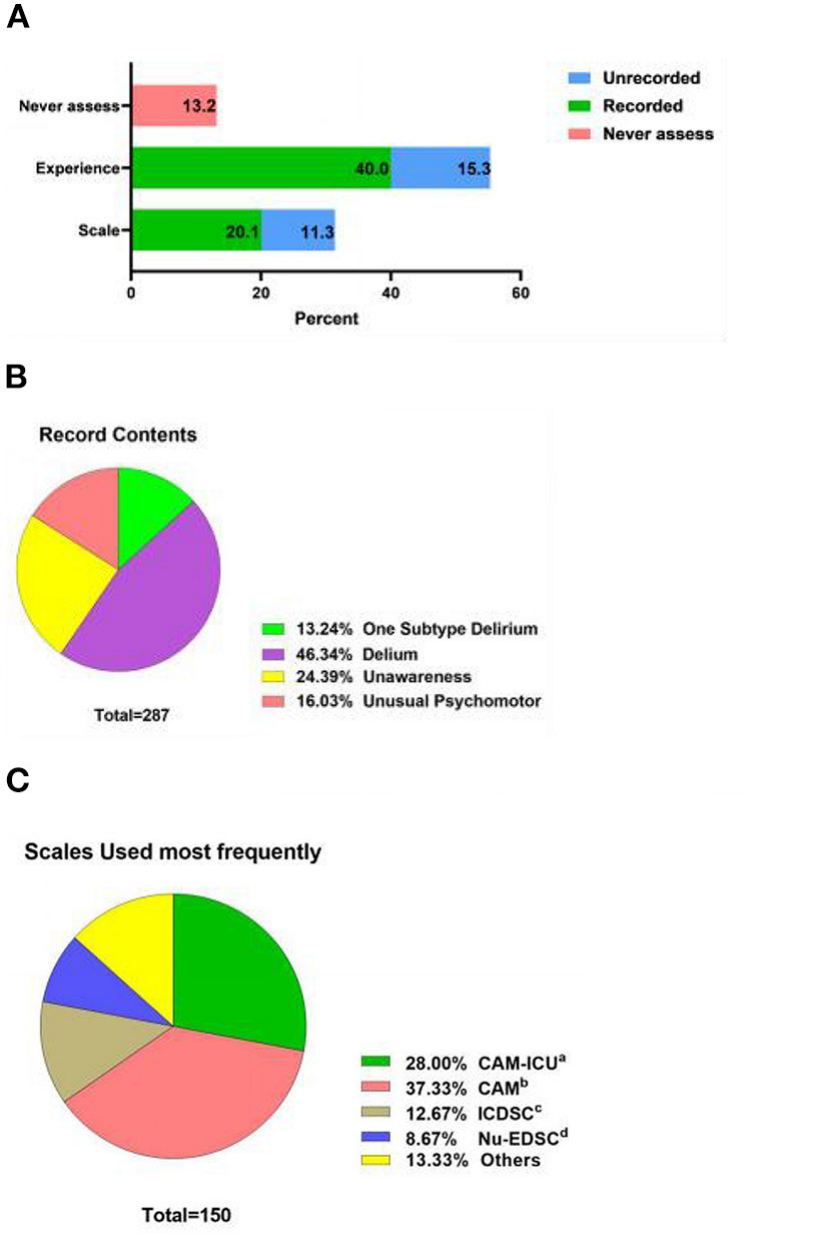
**(A)** Record behaviors regarding delirium for clinical nurses (*n* = 477). **(B)** Record contents regarding delirium for clinical nurses (*n* = 287). **(C)** Delirium assessment scales used most frequently for clinical nurses (*n* = 150). CAM-ICU, Confusion Assessment Method for Intensive Care Unit; CAM, Confusion Assessment Method; ICDSC, Intensive Care Delirium Screening Checklist; Nu-DESC, Nursing Delirium Screening Scale.

As reported by the participants, the practice scores were 5.17 ± 2.06, and the scoring rate was 51.70%, as shown in Figure 4. More than half assessed delirium through clinical experience, 150 (31.45%) assessed delirium through scales and 63 (13.21%) never assessed it. Details of the percentage and content of records (*n* = 287) are shown in Figures 5A, B. The results of the delirium assessment scales used most frequently are shown in Figure 5C. The Confusion Assessment Method for Intensive Care Unit (CAM-ICU) was the most frequently used delirium assessment scale. The nurses who did not explicitly record delirium in their paperwork (*n* = 127) were further investigated using ancillary multiple-choice questions to determine the reason. In this section, 52.8% of nurses (*n* = 67) reported “no diagnostic tool used”, followed by 44.9% (*n* = 57) who responded that “the department staff routinely records delirium as unconscious/abnormal mental behavior”, 43.3% (*n* = 55) reported that “the doctor did not diagnose delirium definitely”, and 27.6% (*n* = 35) were “not sure if the patient was delirious even after using diagnostic tools”. Regarding delirium treatment, 93.3% of nurses cooperated with a physician, 44.7% worked with nursing partners, 31.2% preferred to ask for a psychiatrist's help, 23.1% consulted the psychiatrist and treated the patient by themselves, only 1.9% to resolved the problem independently, and 2.3% ignored it.

Subtypes practice seemed to be worse. In this survey, more than half (250, 52.4%) of nurses occasionally or sometimes assessed delirium subtypes, and 143 (30.0%) never assessed delirium subtypes. For nurses who seldom or never assessed for subtypes (*n* = 393), ancillary multiple-choice questions were used to investigate the reason for poor assessment behavior. A total of 299 nurses (76.1%) acknowledged “deficient knowledge of subtypes”, 277 (57.8%) attributed poorness to “do not know any subtype measurement”, and 154 (39.1%) selected “do not know how to use delirium subtype assessment tools”. Only 35 (8.1%) thought “it was not necessary to assess delirium subtypes”, and 30 (7.6%) selected “other reasons”, with filled-in-the-blank explanations including “not in the scope of nurses' work,” “less attention,” and “not encountered by the department.”

A total of 332 (69.60%) participants thought that hyperactive delirium was the most common subtype in daily work, followed by 68 (14.3%) who reported a mixed subtype, 31 (6.5%) who reported hypoactive, and 46 (9.6%) reported that they were unable to distinguish subtypes. Among the nurses (*n* = 431) who were able to distinguish subtypes, the survey found that the majority (379, 87.9%) assessed subtypes through clinical experience, followed by consultation with colleagues (147, 34.1%), with the help of scales (112, 26.0%), and others (22, 5.1%). Further investigation revealed that the most commonly used delirium subtype assessment scale among the 112 participants surveyed using fill-in-the-blank questions was CAM-ICU (32, 28.6%), followed by Confusion Assessment Method (21, 18.8%), Intensive Care Delirium Screening Checklist (6, 5.4%), Nursing Delirium Screening Scale (3, 2.7%), and Richmond Agitation-Sedation Scale (3, 2.7%), and 48 were invalid answers (such as “I don't know” or “I have not used any scale” in the blank box) accounting for 42.9%.

### 3.5. Factors affecting the scores of clinical nurses regarding delirium and subtype assessment

The total KAP score of 477 clinical nurses was 60.01 ± 6.98, with a scoring rate of 73.18%. We conducted one-way ANOVA analysis, Pearson's correlation analysis, and stepwise multiple linear regression to explore the factors affecting scores. The 11 questions on demographics were set as the independent variables, and the scores of each section and the KAP were set as the dependent variables. One-way ANOVA analysis showed that age, clinical work department, title, familiarity with delirium, familiarity with delirium subtypes, delirium training, and delirium subtype training influenced the KAP scores regarding clinical nurses' delirium and subtype assessment, as detailed in Table 1. The results of stepwise multiple linear regression are shown in Table 4. The variables that affected the total KAP score were delirium familiarity (β = 0.310), work department (β = −0.160), technical title (β = −0.142), and education (β = −0.106). Work department was the most influential fact on the knowledge scores. The familiarity of delirium had an impact on the attitude dimension of 23.3%. In the practice dimension, the familiarity of delirium (β = 0.263), work department (β = −0.162), familiarity of subtypes (β = 0.176), technical title (β = −0.144), and training of subtype (β = 0.127) were significantly associated with the practice score. The results of Pearson's correlation analysis of each section score and the KAP scores are shown in Supplementary material 3.

**Table 4:** Multiple linear stepwise regression analysis of delirium and subtype assessment for clinical nurses (*n* = 477).

	**Variables**	***R***	***R* ^2^**	**Adjusted *R*^2^**	**B**	**Std. error**	**β**	***t***	**95 CI**	***F***	***P*-value**
Knowledge score	Constant	13.336	0.743	17.948	11.876 to 14.796
	Work department^a^	0.215	0.046	0.044	−0.341	0.035	−0.182	−4.027	−0.507 to −0.174	22.968	<0.001
	Group leader^b^	0.235	0.055	0.051	1.097	0.374	0.138	2.936	0.363–1.832	13.889	0.003
	Age^c^	0.270	0.073	0.067	−0.650	0.205	−0.152	−3.171	−1.052 to −0.247	12.351	0.002
	Education^d^	0.284	0.081	0.073	0.703	0.349	0.090	2.015	0.017–1.388	10.339	0.042
Attitude score	Constant	37.226	0.94	39.740	35.385–39.067
	Familiarity of delirium	0.233	0.054	0.052	1.419	0.272	0.233	5.213	0.884–1.953	27.174	<0.001
Practice score	Constant	2.955	0.445	6.647	2.082–3.829
	Familiarity of delirium	0.469	0.220	0.219	0.656	0.138	0.263	4.759	0.385–0.927	134.185	<0.001
	Work department^a^	0.511	0.261	0.257	−0.222	0.058	−0.162	−3.850	−0.335 to −0.108	83.548	<0.001
	Familiarity of subtypes	0.533	0.284	0.279	0.427	0.131	0.176	3.253	0.169–0.684	62.473	0.001
	Technical title^e^	0.550	0.302	0.296	−0.353	0.096	−0.144	−3.683	−0.541 to −0.165	51.049	<0.001
	Training of subtypes^f^	0.562	0.316	0.308	0.727	0.236	0.127	3.077	0.263–1.191	43.465	0.002
Final score	Constant	51.929	2.138	24.290	47.728–56.130
	Familiarity of delirium	0.361	0.130	0.128	2.613	0.375	0.310	6.973	1.877–3.349	71.139	<0.001
	Work department^a^	0.404	0.163	0.160	−0.743	0.209	−0.160	−3.547	−1.155 to −0.332	46.279	<0.001
	Technical title^e^	0.419	0.175	0.170	−1.173	0.367	−0.142	−3.200	−1.894 to −0.453	33.551	0.001
	Education^d^	0.430	0.185	0.178	1.999	0.837	0.106	2.387	0.353–3.645	26.837	0.017

a Dummy variable (1, general ICU; 2, specialized ICU; 3, orthopedics; 4, cardio-thoracic surgery; 5, anesthesiology; 6, geriatrics).

b Dummy variable (0, no; 1, yes).

c Dummy variable (1, 20–29 years; 2, 30–39 years; 3, 40–49 years; 4, 50–59 years).

d Dummy variable (1, associate degree; 2, bachelor degree; 3, master degree and doctor degree).

e Dummy variable [1, primary; 2, primary (supervisor); 3, moderate; 4, senior].

f Dummy variable (0, no; 1, yes).

B is unstandardized coefficients, β is standardized coefficients, CI is 95.0% confidence interval.

## 4. Discussion

As the first caregiver of hospitalized patients, the knowledge-attitude-practice status among nurses affects standardize practice and assessment (55). The barriers to assessment and practice, the influencing factors, and details in practice progress also need to be considered. Previous studies have shown that there are significant differences in the predisposing factors, etiology, treatment and outcomes among each subtype (56–58). It is necessary to clarify nurses' KAP status in subtype assessment to make subtype management protocols in the future. However, most studies focused on the epidemiology of subtype rather than the individual's ability, and there have been few studies exploring nurses' KAP status regarding assessment of delirium subtypes, especially in attitude and practice (59–61). Therefore, we developed a KAP questionnaire for delirium and subtype assessment, which has several question types and ancillary questions, to explore the details. Our research group adhered strictly to the methodology of questionnaire development to ensure the scientific construction of the instrument. The questionnaire we developed has good reliability and validity. To the knowledge of the authors, the KAP level of delirium subtypes is explored first in this study. The survey investigated clinical nurses' KAP status, knowledge sources, barriers to delirium and subtype assessment and self-evaluation and practice details (including assessment instruments used and recording content). This study expands on previous findings by demonstrating that there is disparity between the KAP level regarding delirium and subtype assessment between nurses in different departments, the existence of inadequate knowledge and experience-driven practice, and a potential role for administrative support to improve delirium and subtype management.

The most important findings of this multicentre survey on delirium and subtype assessment among Chinese clinical nurses can be summarized as follows: (1) the KAP level was not too insufficient but still needed to improve, the attitude status was positive while the knowledge was inadequate and practice was imperfect; (2) the KAP level varied significantly between departments, with the ICU nurses ranking highest; (3) nurses' inadequate knowledge of delirium and delirium subtype assessment was a significant individual-level barrier to delirium and subtype assessment; (4) misunderstanding existed about delirium and subtype assessment tools; (5) the main knowledge source was the accumulation of experience and communication in daily clinical work, while nurses desired systematic training; (6) the delirium assessment work in the department was less satisfactory because of lack of the workable protocols, scientific training, and documentation standards regarding delirium assessment and management; (7) delirium and subtype assessment is currently based on experience; (8) most nurses manage delirium in collaboration with physicians and rarely independently manage it; (9) assessment practice for delirium subtype is worse than for delirium because of the lack of subtype knowledge and the availability of subtype assessment tools.

In the knowledge section, 37.5% of the participants only chose increased activity as a clinical manifestation of delirium. This finding indicated that more nurses considered increased activity to be a clinical manifestation of delirium, but decreased activity was not, which may be related to the ignorance or inaccuracy of hypoactive delirium assessment. Delirium subtypes were assessed by 70% of nurses in their clinical work, and 69.6% of nurses considered hyperactivity to be the most common symptom observed in their daily work. However, previous studies have shown that hyperactive delirium accounted for approximately a quarter of delirium patients while hypoactive and mixed delirium accounted for more. Hyperactive was noticed frequently, which could likewise reflect the findings of Marcantoni's study that hypoactive delirium was more likely to be overlooked by health care staff (4). This finding is also related to the results of Inouye's study, which reflected that hypoactive delirium is one of the independent risk factors for underrecognition by nurses (62). Sun has also reported that only 17.6% of Chinese ICU nurses can assess hypoactive delirium accurately (18). A review also mentioned that the docile patients may be overlooked because they pay less attention to the care provider (63). Moreover, the most commonly used delirium subtype assessment scale among the 112 participants surveyed was the CAM-ICU (32, 28.6%), followed by the CAM (21, 18.8%), ICDSC (6, 5.4%), Nu-EDSC (3, 2.7%), RASS (3, 2.7%), and other/invalid answers accounted for 42.9% of responses. In fact, only the RASS can be used to assess subtypes; the others are used to assess only delirium (32, 64). Additionally, we found that 59.7% of nurses did not believe the assessment results, which can be explained by the fact that nurses are unfamiliar with the delirium assessment tools and are less confident in their abilities, similar to the finding of Yue's study (65). Combining the results regarding delirium and subtype assessment tools in knowledge and practice dimensions, we speculate that there is misunderstanding and unfamiliarity in subtype assessment tools among nurses. Additionally, nurses misunderstand (75.1%) delirium prevention and restraint, which is associated with that a retrospective secondary analysis of 4,200 patients in Iran showing that increased delirium risk was associated with exposure to physical restraint application (66). However, most nurses know about the adverse outcomes and high-risk factors for delirium, which is the same as Xie's survey findings among orthopedic professional nurses and Xu's among ICU nurses (59, 61). Overall, the knowledge level is not sufficient, which affects practice, as the individual-level barrier results in this study showed. Nurses need additional delirium education in further.

This study investigated the knowledge sources to explain the knowledge status, and found that the main knowledge sources are work experience accumulation (69.4%), communication with colleagues (57.2%) and knowledge learned in school (43.6%), which is also similar to the findings of Xie's study (61). In this study, the first three choices are expected among nurses, including academic conferences (68.8%), academic training (51.6%), and knowledge handbook or poster (46.3%), which are systematic, standardized, specialized, and visualized training. This is also similar to the results of Wang's qualitative research (67). Previous studies focus on delirium training. Baessler et al.'s research found a different effectiveness on delirium knowledge levels among medical students before and after different teaching method interventions (68). Wang developed a training program protocol for the evaluation of delirium in the Chinese ICU (67). Although previous studies have shown the effectiveness of training and several guidelines (13, 33, 42) have clarified the importance of delirium assessment and management, exercisable training is still needed (61, 69). Possible explanations for this are that the training program is rarely applied in other departments except the ICU and there is a lack of training content about subtype assessment and management, especially the differences in the clinical manifestations and management.

Another way to look at it is that the organizational lack of important awareness may lagging behind that of the individual level. We clarified the individuals' awareness, that is, the attitude dimension scoring rate (83.94%), which means that most clinical nurses have a strongly positive attitude toward delirium and subtype assessment. This finding is similar to Xie's study, with an attitude scoring rate of 80.85%. In our study, the strongly positive attitude of subtypes was added, which means that nurses are beginning to recognize that there are certain benefits to managing different delirium subtypes, such as increased efficiency of care and human resource savings. This study showed that “failure to incorporate delirium assessment into routine tasks” was chosen by nearly 70% of nurses. In Xie's opinion, this may be related to the department not taking this as the nurse professional assessment index (61). Furthermore, this study indicated that 26.6% of nurses performed delirium assessments but did not explicitly record “delirium” in the nursing paperwork. The lack of knowledge, acquiescent record rules and dependence on doctors' diagnoses were all selected by half of the participants. Additionally, 93.3% of nurses managed delirium with a physician rather than by themselves. All of the above may be related to the lack of self-confidence in delirium management, suggested by Yue's study (65). An electronic survey in the Netherlands also pointed out that compared with physicians, nurses were less confident with delirium screening tools and were less convinced that delirium can be prevented (39). Moreover, the medical environment in mainland China is responsible. For health care professionals in China, the current management of delirium is still based on medical orders, sedatives for hyperactive symptoms and nothing for hypoactive symptoms as usual, and nurse are responsible for executing these orders. Xie also mentioned the unclear division of labor between physicians and nurses (61). As the practice dimension reflects, a total of 414 (86.79%) nurses assessed delirium in their clinical work, with 264 (55.35%) reporting clinical experience as a basis and 150 (31.4%) using diagnostic scales. Our findings reflected that although most nurses were aware of diagnostic tools such as the CAM-ICU, they did not use standardized tools and still relied on empirical judgements, which Yue also reported (65). Thus, further research about training, including delirium and subtype assessment and management, is needed, and organizational support is worth considering, including clearer responsibility regarding delirium and subtype assessment among medical staff and the recording of assessments and symptoms in patient records, to change the current experience-driven methods. In addition, more studies can be conducted to identify hypoactivity and reduce the rate of missed diagnosis by combining tools such as risk prediction models and scales with daily work systems.

This study confirms that the working department is one of the essential factors in delirium and subtype assessment. In this survey, we recruited a sample of 477 nurses from six departments with a higher delirium incidence than other departments to explore the impact on nurses' KAP level regarding delirium and subtype assessment from a departmental perspective. Through multiple stepwise regression analysis, we found that department had a more significant effect on the total score, knowledge score, and practice score, but not the attitude score. The investigation showed that the general ICU had the highest total score. This may be because the medical environment of the general ICU has too many pressure sources that can cause delirium, so ICU nurses are most aware of delirium happening (70). ICU patients have worse physiological function, which can increase the incidence of delirium in the ICU and alert the medical staff (5). Thoracic surgery and orthopedics scored essentially the same, probably because the high incidence of delirium in postoperative patients in cardiac surgery and orthopedics has raised the concern of nurses (25, 71). However, nurses in anesthesiology and geriatrics had lower KAP scores, probably because delirium mainly occurs within 1–3 days after surgery (72), but in this survey, the cases related to anesthesia were short-term. Perhaps patients were not on anesthesiology equipment when delirium occurred. Age is a risk factor for delirium (12). Since geriatric departments in Chinese health care institutions mostly admit elderly patients with chronic diseases rather than with consciousness disorders, delirium has not yet become a routine assessment task and delirium screening tools have not been widely used (73). Thus, it is necessary to explore the differences in delirium between departments.

Moreover, common delirium and subtype measurements or instruments may be adjusted according to clinical work in China mainland. Some nurses mentioned that the items of individual delirium assessment tools were difficult to use, which is consistent with Yue's study (65). This may be because delirium is inherently a transient, acute, and transitory change in consciousness. It is difficult for nurses to monitor the patient's consciousness because of a busy clinical schedule and stretched human resources in the Chinese clinical situation. In addition, most of the delirium assessment tools currently used in the Chinese health care environment are derived from original English publications. Perhaps the language change is still not localized despite the cultural adjustment steps in derivation. Some nurses also mentioned that they were not confident in their assessments due to a lack of proficiency. Therefore, further research regarding cultural adjustment of instruments is needed, or consideration should be given to adding explanations of the items' connotations in order that clinical nurses can better understand and improve practices. Training on instruments should be conducted, ensuring that each nurse can correctly use the instruments to diagnose delirium and subtypes in the condition of effectiveness accurately.

There were some limitations to this study. First, 10 experts agreed well with each other in the development of the KAP questionnaire regarding delirium and subtype assessment. We think that this may be related to the fact that only one expert was a physician from the ICU and other experts majored in nursing. Second, there were no reverse-scored questions in the questionnaire because of the complexity of question types and contents. Moreover, this study was conducted in only three Chinese provinces, and a wide geographical area and large sample should be considered. Perhaps a qualitative study could obtain more details on the obstructive factors of subtype assessment. Finally, the answer to the assessment tools had too many invalid responses, indicating possible selection bias.

## 5. Conclusion

According to the present study results, the total KAP scoring rate of Chinese clinical nurses regarding delirium and subtype assessment was barely acceptable, while the attitude status was positive, but the knowledge and practice status needed to be changed. Inadequate knowledge regarding delirium subtypes and unfamiliarity regarding assessment tools are the most influential barriers to practice at the individual-level, and experience still drives nurses' assessments of delirium and subtypes. Adding the delirium assessment into routine tasks should be considered. Further, systematic training and organizational support for nurses are recommended. We also suggest more exploration among different departments should be discussed in future. Additionally, the assessment tools need to be revised for clinical use.

## Data availability statement

The raw data supporting the conclusions of this article will be made available by the authors, without undue reservation.

## Ethics statement

The studies involving human participants were reviewed and approved by Second Hospital of Chongqing Medical University. The patients/participants provided their informed consent to participate in this study before the formal survey beginning. The informed consent was shown in the first page of the e-questionnaire.

## Author contributions

WZ: conceptualization, data curation, formal analysis, investigation, methodology, project administration, writing—original draft, and visualization. QZ and MH: conceptualization, methodology, project administration, validation, and writing—review and editing. CZ: data curation, investigation, supervision, and project administration. HZ, LY, and TW: investigation. XG: conceptualization, methodology, project administration, validation, writing—review and editing, supervision, project administration, funding acquisition, and resources. All authors contributed to the article and approved the submitted version.
