# Corrigendum: Knowledge, attitude, and practice toward delirium and subtype assessment among Chinese clinical nurses and determinant factors: A multicentre cross-section study

**DOI:** 10.3389/fpsyt.2024.1483648

**Published:** 2024-09-03

**Authors:** Wen Zhou, Qiulan Zheng, Miao Huang, Chuanlai Zhang, Huan Zhang, Li Yang, Taiqin Wu, Xiuni Gan

**Affiliations:** ^1^ Department of Nursing, Second Affiliated Hospital of Chongqing Medical University, Chongqing, China; ^2^ The Second Department of Nursing School, Chongqing Medical University, Chongqing, China; ^3^ Department of Intensive Care Unit, Second Affiliated Hospital, Chongqing Medical University, Chongqing, China

**Keywords:** delirium, delirium subtypes, measurements, nursing assessment, assessment frequency

In the published article, there was an error in affiliations 1 and 2.

The affiliations read: “1 The Second Department of Nursing School, Chongqing Medical University, Chongqing, China”

“2 Department of Nursing, Second Affiliated Hospital, Chongqing Medical University, Chongqing, China”

They have been corrected to: “1 Department of Nursing, Second Affiliated Hospital of Chongqing Medical University, Chongqing, China

“2 The Second Department of Nursing School, Chongqing Medical University, Chongqing, China”

The authors apologize for this error and state that this does not change the scientific conclusions of the article in any way.

The original article has been updated.

