# Supplementary material for: Knowledge, attitude, and practice toward delirium and subtype assessment among Chinese clinical nurses and determinant factors: A multicentre cross-section study
Source: Front Psychiatry. 2023 Feb 1;13:1017283. doi: 10.3389/fpsyt.2022.1017283 (PMC9929153; doi:10.3389/fpsyt.2022.1017283)
Supplement: Supplementary file 2 [file Data_Sheet_2.DOCX]

## Supplementary file 2 - Knowledge, attitude, practice (KAP) questionnaire regarding delirium and subtypes assessment among nurses

| Questionnaire | | |
| --- | --- | --- |
|  |  |  |
| Dimension 1:Knowledge | | |
|  | 1.1 | Judgment: Delirium is an acute reversible mental disorder caused by various diseases. |
|  | 1.2 | Multiple Choice: What are the risks of delirium?  ⑴Increased mortality;  ⑵Prolonged hospitalization;  ⑶Increased hospitalization costs;  ⑷Residual long-term perceptual impairment;  ⑸Unawareness |
|  | 1.3 | Multiple Choice: Which groups are at high risk for delirium?  ⑴ICU patients; ⑵Post-operative patients; ⑶Elderly patients; ⑷Palliative care patients; ⑸Unawareness |
|  | 1.4 | Multiple Choice: What are the risk factors for delirium?  ⑴Patient factors: age, whether combined with underlying diseases, etc.;  ⑵Drug factors: sedative drugs, analgesic drugs, etc.;  ⑶Surgical factors: type of surgery, postoperative pain, etc.;  ⑷Environmental factors: lights, machine alarms, etc.;  ⑸Psychological factors: anxiety, depression, sense of stress, etc.;  ⑹Nursing factors: restraint, therapeutic operations, etc.;  ⑺Unawareness |
|  | 1.5 | Multiple Choice: What are the clinical features of delirium?  ⑴Inability to concentrate;  ⑵Disorganized thinking;  ⑶Increased activity;  ⑷Decreased activity;  ⑸Altered state of consciousness;  ⑹Unawareness |
|  | 1.6 | Multiple Choice: Which of the following tools are delirium assessment tools?  ⑴DSM-5; ⑵ICD-10; ⑶CAM; ⑷CAM-ICU; ⑸ICDSC; ⑹Unawareness |
|  | 1.7 | Multiple Choice: What are the management measures after the occurrence of delirium?  ⑴Treatment of the cause;  ⑵Intensive management;  ⑶Early activity;  ⑷Emphasis on sleep management;  ⑸Targeted treatment according to different subtypes of delirium;  ⑹Unawareness |
|  | 1.8 | Multiple Choice: Key strategies to prevent and reduce delirium include which?  ⑴Identify and modify risk factors that lead to delirium;  ⑵Early detection of patients at risk for delirium;  ⑶Pay attention to patients' sleep management;  ⑷Help patients at risk for delirium to perform early rehabilitation activities;  ⑸Take timely restraint measures for patients with delirium;  ⑹Unawareness |
|  | 1.9 | Multiple choice: What is correct about the following clinical manifestations of each subtype of delirium?  ⑴Hyporactive delirium is characterized by emotional poverty, indifference, drowsiness, and decreased reactivity;  ⑵Hyperactive delirium is characterized by agitation, anxiety, and attempts to catch extubation;  ⑶Mixed delirium shows fluctuations in agitation and quiet symptoms;  ⑷Unawareness |
|  | 1.10 | Multiple Choice: What is correct about the following adverse outcomes for each subtype of delirium?  ⑴Patients with increased activity delirium are relatively more likely to have adverse events such as falls, bed falls, and catheter extraction accidents;  ⑵Patients with increased activity delirium are less likely to be detected by health care personnel;  ⑶Patients with decreased activity delirium are more likely to have stress injuries;  ⑷Decreased activity delirium is less likely to be noticed by health care personnel and has more severe effects on patients;  ⑸It is not clear and cannot be judged |
|  | 1.11 | Multiple Choice: Which of the following are delirium subtype assessment tools?  ⑴ICDSC; ⑵RASS; ⑶DMSS; ⑷MDAS; ⑸Unawareness |
| Dimension 2：Attitude | | |
|  | 2.1 | Scoring: How important do you think nursing care is in preventing and recovering delirium? |
|  | 2.2 | Scoring: Do you think clinical nurses should undertake the identification of delirium and delirium subtypes? |
|  | 2.3 | Scoring: Do you think clinical nurses should know delirium and delirium subtypes? |
|  | 2.4 | Scoring: Do you think your knowledge of delirium and delirium subtypes can meet clinical needs? |
|  | 2.5 | Scoring: Are you interested in knowledge about delirium and delirium subtypes? |
|  | 2.6 | Scoring: Do clinical nurses need to learn about delirium and its subtypes actively? |
|  | 2.7 | Scoring: Do you think clinical nurses must receive systematic training on knowledge related to delirium and delirium subtypes? |
|  | 2.8 | Scoring: Do you think conducting a delirium subtype assessment in clinical work is necessary? |
|  | 2.9 | Scoring: Do you think it is necessary to develop/introduce delirium subtype assessment tools? |
|  | 2.10 | Scoring: Are you willing to receive training on delirium subtypes? |
|  | 2.11 | Multiple Choice: Which of the following types of delirium have you heard of?  ⑴Hyperactive delirium;  ⑵Hypoactive delirium  ⑶Mixed delirium;  ⑷Quiet delirium;  ⑸Excited delirium;  ⑹Depressed delirium;  ⑺No motor delirium;  ⑻Mixed delirium;  ⑼Other ___________ (please fill in);  ⑽ none of the above have been heard of |
|  | 2.12 | Multiple Choice: What are your requirements for delirium assessment tools?  ⑴Accurate assessment results;  ⑵Reasonable assessment time;  ⑶Reasonable frequency of assessment;  ⑷Clear and easy to understand the text;  ⑸Concise and clear forms;  ⑹Other ___________ (please fill in) |
|  | 2.13 | Multiple Choice: At the individual level, what are the current barriers to early delirium recognition for nurses?  ⑴Insufficient knowledge base of delirium;  ⑵Insufficient mastery of delirium assessment methods;  ⑶Insufficient proficiency in the use of delirium assessment scales;  ⑷Delirium assessment increases workload;  ⑸Nurses are not confident enough in terms of their ability to assess delirium and do not trust the results of their assessment;  ⑹Nurses are busy with clinical work and lack time to conduct delirium assessments;  ⑺Nurses do not cooperate sufficiently with physicians;  ⑻Other _________________(please fill in) |
|  | 2.14 | Multiple Choice: At the organizational level, what are the current barriers to nurses' early identification of delirium?  ⑴The department/hospital does not have process specifications related to delirium assessment;  ⑵The department/hospital does not conduct training related to delirium assessment;  ⑶The department does not routinely conduct delirium assessments;  ⑷The department does not provide delirium assessment tools;  ⑸The department's human resource allocation is inadequate;  ⑹Other _________________(please fill in) |
|  | 2.15 | Multiple Choice: In your opinion, what are the barriers to delirium subtype assessment?  ⑴There is no significant difference in the clinical manifestations of each delirium subtype;  ⑵There is no significant difference in the management measures of each delirium subtype;  ⑶There is no significant difference in the prognostic impact of each delirium subtype;  ⑷Nurses are busy with clinical work, and delirium subtype assessment will increase the workload of nurses;  ⑸Nurses do not have enough knowledge of delirium subtypes and assessment methods;  ⑹Nurses lack objective delirium subtype assessment tools;  ⑺Delirium assessment work is still immature, and subtype assessment work is not carried out at all;  ⑻The department/hospital does not focus on this part of the delirium subtype assessment at present and does not request the staff to assess the delirium subtype;  ⑼Other __________(please fill in) |
|  | 2.16 | Single Choice: How well do you think delirium assessment is done in the section you work in? (If it is convenient, please briefly describe the problems that exist)  ⑴Very well done;  ⑵Basically well done, but still some details are not enough __________;  ⑶Not well done, still many problems ___________;  ⑷No delirium assessment work at all |
| Dimension 3:Practice | | |
|  | 3.1 | Single Choice： In your daily clinical work, do you assess delirium?  ⑴Always; ⑵Often; ⑶Sometimes; ⑷Sometimes; ⑸Never |
|  | 3.2 | Single Choice： How do you assess and document delirium in your clinical work?  ⑴Assessed by diagnostic scales and recorded;  ⑵Assessed by diagnostic scales but not recorded;  ⑶Assessed by clinical experience only and recorded;  ⑷Assessed by clinical experience only and not recorded;  ⑸ Did not assess delirium |
|  |  | 3.2.1 Single Choice: What do you record about delirium? (⑴ or ⑶ was selected for 3.2)  A. "Patient has delirium of type xxx."  B. "Patient has delirium."  C. "Patient has confusion."  D. "Patient has Behavioral mental abnormalities."  E. Other, please describe _________________ |
|  |  | 3.2.1.1 Multiple Choice: Why don't you record this as "patient has delirium"? ( C or D was selected for 3.2.1)  (1) No delirium diagnostic tool was used for assessment;  (2) Diagnostic tool was used but still not sure if the patient had delirium;  (3) Physician did not make a diagnosis of delirium;  (4) In the nursing records of the department, such patients were recorded as "confusion/abnormal mental behavior";  (5) Other _____ (please fill in) |
|  |  | 3.2.2 Single Choice: What is the scale you use most frequently? (⑴ or ⑵ was selected for 3.2)  A.CAM; B.CAM-ICU; C.ICDSD; D.Nu-DESC; E.Other _____ (please fill in) |
|  | 3.3 | Multiple Choice: How do you usually solve delirium problems when you encounter them in your daily clinical work?  ⑴Discuss with doctors to solve the problem;  ⑵Discuss with other nurses to solve the problem;  ⑶Ask psychiatrists/psychologists to solve the problem;  ⑷ Consult psychiatrists/psychologists;  ⑸ Solve the problem independently;  ⑹Other ___________(please fill in) |
|  | 3.4 | Single Choice: In your daily clinical work, do you assess your patients' type of delirium (delirium subtype)?  ⑴always; ⑵often; ⑶sometimes; ⑷occasionally; ⑸ never |
|  |  | 3.4.1 Multiple Choice: Please tell me why you do not assess/are less likely to assess delirium subtypes? (⑶ or ⑷ or ⑸ was selected for 3.4)  A. Do not know about delirium subtypes;  B. Do not have delirium subtype assessment workers  C. Do not know how to use delirium subtype assessment tools;  D. Do not think the assessment is necessary;  E. Other __________ (please fill in) |
|  | 3.5 | Single Choice: In your daily clinical work, which of the following delirium patients are more common?  ⑴Manic patients: increased speech, restlessness, and uncontrolled behavior;  ⑵Quiet patients: slower movements, reduced speech, and lowered speaking volume;  ⑶Mixed patients: alternation of the above two manifestations;  ⑷I cannot distinguish between the above types of delirium patients |
|  |  | 3.5.1 Multiple Choice: How would you assess the patient's delirium subtype? (⑴or ⑵ or ⑶ was selected for 3.5)  A. By clinical experience;  B. By assessing with the help of specific scales;  C. By consulting with colleagues;  D. Other ______ (please fill in) |
|  |  | 3.5.1.1 Fill in the blanks: I am using scale to assess delirium subtypes. (B was selected for 3.5.1) |
| Dimension 4：Knowledge sources | | |
|  | 4.1 | Scoring: Does the knowledge you learned in school about delirium meet the needs of your current clinical work? |
|  | 4.2 | Single Choice: Have you ever participated in delirium-related knowledge training?  ⑴Yes; ⑵No |
|  |  | 4.2.1Single Choice: If you have participated in the training, at the end of the training, did you pass the training assessment?  A. all passed;  B. can pass about 80% or more of the assessment;  C. can pass about 50%-80% of the assessment;  D. can pass about 30%-50% of the assessment;  E. can only pass about 30% of the assessment;  F. no assessment session set |
|  |  | 4.2.2 Multiple Choice: Which of the following categories/does the knowledge training you attended belong to?  A. Hospital level physician lectures (led by physicians/medical department, etc.);  B. Hospital level nurse lectures (led by nurses or nursing department, etc.);  C. Department level physician lectures (led by physician teaching team leader/director, etc.);  D. Department level nurse lectures (led by nurse teaching team leader/nurse manager, etc.);  E. Outbound training and learning;  F. Participation in academic conferences;  H. Personal initiative to learn relevant knowledge.  I. Other ________(please fill in) |
|  | 4.3 | Multiple Choice: Your knowledge of delirium and delirium subtypes comes primarily from the following sources? |
|  | 4.4 | Multiple Choice: In what ways would you most like to enhance your knowledge about delirium and delirium subtypes? |
|  |  | 4.3&4.4 Options:  ⑴ Study at school;  ⑵ Academic conferences and lectures;  ⑶ Relevant study classes;  ⑷ Self-study (due to personal interest or work needs);  ⑸ Work experience accumulation;  ⑹ Exchange among colleagues;  ⑺ Relevant media reports;  ⑻ Consult relevant experts;  ⑼ Brochures and publicity wall posters;  ⑽ Others _______________(please fill in) |
|  | 4.5 | Multiple Choice: What are you most looking forward to learning about delirium and delirium subtypes? (Select up to 5 items)  ⑴ Definition of delirium;  ⑵ Monitoring and diagnosis of delirium;  ⑶ Risk factors and etiology of delirium;  ⑷ Prevention and management of delirium;  ⑸ Definition and clinical manifestations of delirium subtypes;  ⑹ Assessment methods and assessment tools of delirium subtypes;  ⑺ Nursing measures and nursing priorities of delirium subtypes;  ⑻ Other ______________(please fill in) |
