# Supplementary material for: Knowledge, attitude, and practice toward delirium and subtype assessment among Chinese clinical nurses and determinant factors: A multicentre cross-section study
Source: Front Psychiatry. 2023 Feb 1;13:1017283. doi: 10.3389/fpsyt.2022.1017283 (PMC9929153; doi:10.3389/fpsyt.2022.1017283)
Supplement: Supplementary file 3 [file Data_Sheet_3.docx]

Supplementary File 3 Pearson’s correlation analysis of delirium and subtypes assessment for clinical nurses (n=477)

|  | | Age | Gender | Clinical Working Years | Work Department | Education | Technical title | Position | | | | | Familiarity of delirium | Familiarity with delirium subtypes | Training of delirium | Training of delirium subtypes |
| --- | --- | --- | --- | --- | --- | --- | --- | --- | --- | --- | --- | --- | --- | --- | --- | --- |
|  |  |  |  |  |  |  |  | Duty  Nurse | Group Leader | Clinical Instructor | Specialist nurse | Head nurse |  |  |  |  |
| Knowledge  Score | r | -0.131 | -0.012 | -0.088 | -0.2 | 0.085 | -0.085 | 0.079 | 0.101 | -0.009 | -0.054 | -0.027 | 0.133 | 0.089 | 0.132 | 0.045 |
|  | p | 0.002 | 0.398 | 0.027 | ＜0.001 | 0.031 | 0.032 | 0.043 | 0.013 | 0.423 | 0.119 | 0.28 | 0.002 | 0.026 | 0.004 | 0.331 |
| Attitude  Score | r | -0.05 | -0.025 | -0.002 | -0.107 | 0.077 | -0.067 | 0.052 | 0.024 | -0.027 | ＜0.001 | 0.044 | 0.233 | 0.167 | 0.083 | 0.057 |
|  | p | 0.139 | 0.29 | 0.481 | 0.010 | 0.046 | 0.071 | 0.127 | 0.301 | 0.278 | 0.499 | 0.172 | ＜0.001 | ＜0.001 | 0.070 | 0.213 |
| Practice  Score | r | -0.184 | -0.099 | -0.113 | -0.338 | -0.025 | -0.152 | 0.12 | -0.045 | -0.034 | -0.022 | 0.001 | 0.469 | 0.434 | 0..270 | 0.299 |
|  | p | ＜0.001 | 0.016 | 0.007 | ＜0.001 | 0.296 | ＜0.001 | 0.004 | 0.164 | 0.227 | 0.313 | 0.493 | ＜0.001 | ＜0.001 | ＜0.001 | ＜0.001 |
| Total  Score | r | -0.143 | -0.052 | -0.07 | -0.258 | 0.083 | -0.128 | 0.105 | 0.045 | -0.033 | -0.028 | 0.021 | 0.361 | 0.285 | 0.193 | 0.148 |
|  | p | 0.001 | 0.127 | 0.062 | ＜0.001 | 0.035 | 0.003 | 0.011 | 0.163 | 0.234 | 0.268 | 0.325 | ＜0.001 | ＜0.001 | ＜0.001 | 0.001 |
